# What Is the Relationship between Antioxidant Efficacy, Functional Composition, and Genetic Characteristics in Comparing Soybean Resources by Year?

**DOI:** 10.3390/antiox11112249

**Published:** 2022-11-15

**Authors:** Han-Na Chu, Suji Lee, Xiaohan Wang, Chi-Do Wee, Hye-Myeong Yoon, Eun-Suk Jung, Mi-Kyung Seo, Yongseok Kwon, Kyeong-A Jang, Haeng-Ran Kim

**Affiliations:** 1 Department of Agro-Food Resources, National Institute of Agricultural Sciences, Wanju-gun 55365, Republic of Korea; 2 National Agrobiodiversity Center, National Institute of Agricultural Sciences, Jeonju 54874, Republic of Korea

**Keywords:** soybean, cultivation year, isoflavone derivatives, GWAS, correlation study, antioxidants, seed coat color

## Abstract

The aim of this study was to analyze the physiological activity of 48 soybean resources harvested in 2020 to identify the soybean resources’ relationships with individual isoflavone compounds and their genetic properties. These data will subsequently be compared with the research results on soybeans harvested in 2019. Initially, with respect to the physiological activity (6 types) and substances (19 types), this study evaluated the differences between the cultivation year (two years), seed coat color (three colors), and the interaction of the year and seed coat color of soybeans through ANOVA. Among the physiological activities, there were differences in the estrogen, estrogen receptor alpha, and UCP-1 (uncoupling protein-1) activities depending on the cultivation year. Moreover, there were differences in NO (nitric oxide), revealing differences in the ABTS (2, 2′-azino-bis-3ethylbenzo-thiazoline-6-sulfonic acid) and DPPH (2, 2-diphenyl-2-picrylhydrazyl) radical scavenging activities due to the seed coat color and the interaction of the year and seed coat color. Soybeans harvested in 2020 exhibited increased ABTS, DPPH, and NO inhibitory activities and reduced estrogen, estrogen receptor alpha, and UCP-1 activities compared to those harvested in 2019. According to the ANOVA results, eight of the nineteen individual derivatives illustrated yearly differences, while three derivatives displayed differences due to the seed coat color. Secondly, according to the relationship between the efficacy, derivative substances, and genetic properties, it was determined that genistein 7-*O*-(2″-*O*-apiosyl)glucoside (F5) is the individual isoflavone derivative that affected the six types of physiological activity, on which the genome-wide association study (GWAS) showed no significant differences for genetic properties. These results were inconsistent with the 2019 data, where three types of individual compounds, including F5, were proposed as substances that correlated with efficacy and there was a high correlation with genetic properties. Therefore, this study selected B17, B23, B15, B24, and Y7 as excellent varieties that are stable and highly functional in the cultivation environment, producing only small annual differences. The results of this study will be utilized as basic data for predicting soybean varieties and their cultivation, which have high environmental stability under climate variation and properly retain the functional substances and efficacy.

## 1. Introduction

Soybeans (*Glycine max* (L.)), which are well-known due to their various functionalities and nutritional superiority, are mass-produced and have high consumption worldwide. Thus, there has been an increasing interest in the functionality of soybeans [1], and some studies have reported that the incidence rates of breast and prostate cancers, which are common in Western countries, are relatively low in countries with high levels of soybean consumption [2,3,4,5]. In addition, one study reported an inverse relationship between the prevalence of cardiometabolic syndrome (CMS) and soybean consumption by South Korean women of 8.5–17 times per week [6], and another study reported that soybean consumption, instead of animal protein, lowers saturated fat and cholesterol levels and exerts a preventive effect on coronary heart disease (CHD) [7].

Isoflavones, a representative functional substance of soybeans, are physiologically active substances that exhibit antiosteoporosis and anticancer effects, are rich in soybean embryos, and are called phytoestrogens because they are similar to the female hormone estrogen [8]. Thus, soybean consumption by menopausal women reduces the incidence rate of neurodegenerative diseases, and genistein contributes to attenuating isoflurane-induced cognitive impairment [9]. In addition, some studies have identified the antidepressant, antiaging, antidiabetic, antiobesity, anti-inflammatory, and antihypertensive effects of soybeans, in addition to finding that they improve metabolic syndromes [9]. A recent study divided isoflavones, known as one of the important substances related to the functionality of soybeans, into 19 individual components [10,11]. Aglycone-based compounds of genistein, daidzein, and glycitein are the main component groups: genistein derivatives include genistein 7-*O*-(6″-*O*-malonyl)glucoside (6″-*O*-malonylgenistin) and genistein 7-*O*-glucoside (genistin); daidzein derivatives include daidzein 7-*O* (6″-*O*-malonyl)glucoside (6″-*O*-malonyldaidzin) and daidzein 7-*O*-glucoside (daidzin); and glycitein derivatives include glycitein 7-*O* (6″-*O*-malonyl)glucoside (6″-*O*-malonylglycitin) and glycitein 7-*O*-glucoside (glycitin) [12]. Despite various studies being conducted on the relationship between soybeans’ physiological activity and isoflavones, which have focused mainly on single ingredients and utilized limited types of samples (resources) [13,14], few comprehensive interaction studies on the various aspects of soybeans have been conducted. Thus, in our preceding study, we conducted a statistical analysis on the relationship between the efficacy, functional substances (individual isoflavone derivatives), and genetic traits of soybeans by utilizing a large amount of soybean samples (resources) in reference to previous studies. As a result, our prior study explored individual isoflavone derivatives with a high efficacy and relevance and presented related genes by deriving meaningful results, contributing to the enhanced utilization of excellent genetic resources [10].

The recent expansion of the plant-based protein market has resulted in a rapidly increasing demand for soybeans worldwide [15,16]; however, there is an urgent need for the stable production of high-quality and highly functional soybeans due to climate change and the COVID-19 pandemic. In this respect, improvement in soybean breeding and the development of cultivation technology have been focused on to achieve higher levels of soybean production, and various approaches that combine agricultural trait improvement and genetic properties are being utilized [17,18,19,20]. In addition, research has extended to soybean varieties to obtain a stable isoflavone content, suggesting that repeated evaluation in various growing environments is essential for selecting soybean varieties [21]. To this end, Yoon et al. [22] conducted an annual comparison of the isoflavone content, quantity-related traits (number of pods per plant and number of seeds per pod), and major agricultural traits (number of days to flowering and maturation) of Korean native soybean genetic resources to select a genetic resource with little annual variation in its isoflavone content and quantity-related traits.

To overcome the changes in cultivation environments due to climate change and to select soybean resources with a high functionality, this study analyzed the relationship between the efficacy, substances, and genetic resource properties of soybean resources grown in 2019 and 2020 to identify annual variation. Our prior research conducted a study to present the relationship between the efficacy, materials, and genetic traits of 48 soybean resources cultivated and harvested in 2019 [10]. In 2020, this study re-examined which individual isoflavone derivatives and genes were directly involved in the efficacy of the same soybean resources cultivated on the same open field as in 2019 and compared their year-to-year differences. This study selected efficacy, substances, and soybean genetic resources that are less vulnerable to changes in the natural environment, such as climate and precipitation (with little annual variation), to investigate whether the soybean cultivation environment induces their annual variation. These results can be utilized as basic data for selecting highly functional soybean varieties that are less vulnerable to the cultivation environment, as well as selecting and stably maintaining individual derivatives (or indicator substances) that affect their physiological activity.

## 2. Materials and Methods

### 2.1. Cultivation Method for Soybean Resources and Experimental Materials

Among the soybean resources owned by the National Agrobiodiversity Center at the National Institute of Agricultural Sciences, 48 varieties with high diversity (variety, landrace, and breeding line) were selected based on the results of the basic trait investigation registered in the Germplasm Management System [10]. The selected 48 resources and 2 control varieties were sown in June 2020 in the open field of the National Agrobiodiversity Center (located in Jung-dong, Jeonju-si, Jeollabuk-do; latitude/longitude, 35° 4938.37 N/127° 0907.78 E), and the seeds were harvested in September, freeze-dried, and pulverized to form a powder. This powder was utilized as an analysis sample [5]. Based on the Jeonju climate statistics data of the Korea Meteorological Administration, this study calculated the normal based on the meteorological environment during the cultivation period and the last 30 years (1991–2020) to create the data for investigation [22]. A total of 48 resources were classified by seed coat color into 19 types of yellow varieties (Y1–Y19), 25 types of black varieties (B1–B25), 4 types of green-black varieties (G1–G4), with 1 yellow (C1) and 1 black (C2) variety as control varieties (Table 1) [10]. The codes C1–C2, Y1–Y19, B1–B25, and G1–G4 presented in Table 1 are hereinafter abbreviated as C, Y, B, and G.

### 2.2. Preparation of Soybean Seed Extract

Seeds high in flavonoids were extracted for antioxidant activity and cell experiments according to the extraction method described in previous studies, and the final extracts were obtained through concentration and freeze-drying processes. Samples were stored at −80 °C, and stock solution was reconstituted in 100% DMSO for further use [10,23].

### 2.3. Antioxidant Activity Assay

#### 2.3.1. ABTS Radical Scavenging Activity

ABTS radical scavenging activity was measured as follows. First, 7.4 mM of ABTS (Sigma-Aldrich, St. Louis, MO, USA) and 2.6 mM of potassium persulphate (Sigma-Aldrich, St. Louis, MO, USA) were prepared, stirred for 24 h, and set aside for formation of ABTS cations. Subsequently, after the reagent reacted with the extract and was set aside for 30 min, absorbance was measured at 734 nm. In this case, L-ascorbic acid was used as a control [10,24].

#### 2.3.2. DPPH Radical Scavenging Activity

DPPH radical scavenging activity was measured as follows. First, 0.2 mM of DPPH (Sigma-Aldrich, St. Louis, MO, USA) solution was prepared in methanol, reacted with the extract, and set aside for 30 min. Subsequently, absorbance was measured at 734 nm with L-ascorbic acid (Sigma-Aldrich, St. Louis, MO, USA) as a control [10,24].

### 2.4. Cell Potency Assay

#### 2.4.1. Estrogen and ER Alpha Activity

As described by Chu et al. [10], standards and samples were added and cultured according to the method provided by the estrogen enzyme-linked immunosorbent assay (ELISA) kit (K4264-100, BioVision, Milpitas, CA, USA) and estrogen receptor alpha kit (K4267-100, BioVision, Milpitas, CA, USA). Subsequently, the biotin detection antibody working solution and the HRP-streptavidin conjugate (SABC) working solution were sequentially added for reaction, and the 3,3′,5,5′-tetramethylbenzidine (TMB) substrate and stop solution were further added to measure the absorbance at 450 nm.

#### 2.4.2. UCP-1 Activity

As described by Chu et al. [10], standards and samples were added and cultured according to the method provided with the UCP-1 ELISA Kit (CSB-EL025554MO, CUSABIO, Huston, TX, USA). Subsequently, the biotin detection antibody working solution and the SABC working solution were sequentially added for reaction, and the TMB substrate and stop solution were further added to measure the absorbance at 540 nm.

#### 2.4.3. NO Inhibition Activity

According to the method described by Chu et al. [10], RAW 264.7 cells were treated with lipopolysaccharide (LPS) and soybean extract for 24 h. Subsequently, Griess reagent (1% sulfanilamide, 0.1% napthylethylene diamine) and cell supernatant were mixed and reacted in the dark room to measure the absorbance at 520 nm.

### 2.5. Characterization of Isoflavone in Soybean Sources

#### 2.5.1. Chemicals and Reagents

Reference standards of genistein, daidzein, genistin, and daidzin were obtained from Sigma-Aldrich Co. (St. Louis, MO, USA); glycitein, glycitin, sophoricoside, and 6-methoxyflavone (ISTD) were purchased from Extrasynthese (Genay Cedex, Lyon Metropolis, France); 6″-*O*-acetylglycitin from MedChemExpress (Monmouth Junction, Middlesex County, NJ, USA); and 6″-*O*-malonyldaidzin, 6″-*O*-malonylgenistin, 6″-*O*-malonylglycitin, 6″-*O*-acetyldaidzin, and 6″-*O*-acetylgenistin from Synthose, Inc. (Ontario, Canada). LC-MS grade methanol and acetonitrile were offered from Thermo Fisher Scientific, Inc. (Waltham, MA, USA). In addition, formic acid (Junsei Chemical, Tokyo, Japan) was used as solvent additive in isoflavone extraction and separation of isoflavone derivatives in chromatographic system.

#### 2.5.2. Isoflavone Extraction and Solid Phase Extraction (SPE) Process

First, 10 mL of isoflavone extraction solvent (methanol/water/formic acid = 50:45:5, *v*/*v*/*v*) and 1 g of soybean powder sample were mixed, stirred for 30 min, and centrifuged at 3600 rpm for 15 min (LABOGENE 1580R, Bio-Medical Science, Co., Seoul, Korea). Subsequently, the supernatant was filtered using a 0.2 μm polyvinylidene fluoride syringe filter (Thermo Fisher Scientific, Inc., Waltham, MA, USA). Then, 2 mL of methanol and 5 mL of water were flowed for the SPE process. Next, 0.5 mL of the filtrate and the internal standard (6-methoxyflavone, 50 ppm) were separately diluted with 7 mL of distilled water and passed through a C18 SPE cartridge (Hypersep C18 500 mg, Thermo Fisher Scientific, Inc., Waltham, MA, USA). Subsequently, the extract was washed with 5 mL of water, and isoflavones were eluted with 5 mL of 1% formic acid in methanol. The isoflavone extract was concentrated with nitrogen gas, further dissolved with 0.5 mL of an extraction solvent, and filtered via a 0.2 µm filter to conduct ultra-performance liquid chromatography (UPLC)–diode array detection–quadrupole time-of-flight (QToF)/mass spectrometry (MS) analysis [10,11,25].

#### 2.5.3. Isolation and Component Analysis of Individual Isoflavone Derivatives

A CORTECS UPLC T3 analytical column (2.1 mm × 150 mm I.D., 1.6 µm; Waters, Co., Milford, MA, USA) and a CORTECS UPLC T3 VanGuard™ precolumn (2.1 mm × 5 mm I.D., 1.6 µm; Waters, Co.) were used to isolate individual isoflavones from soybean seeds. A UPLC system (ACQUITY UPLC^TM^ system, Waters, Co., Milford, CT, USA) and QToF/MS (Xevo G2-S QToF, Waters MS Technologies, Manchester, UK) were utilized in connection. The detection wavelength ranged from 210 to 400 nm (wavelength at 254 nm for isoflavones), and its sample injection volume and flow rate were set to 1 µL and 0.3 mL/min, respectively. For UPLC analysis, 0.5% formic acid in water for eluent A and 0.5% formic acid in acetonitrile for eluent B were used. The composition of B, which started at 5%, was raised to 25, 50, and 90% at 20, 25, and 30 min, respectively, and it was maintained for 2 min until 32 min. It was then lowered to 5% until 35 min, after which it stabilized by maintaining its state until 40 min. For isoflavone structure identification, MS was performed on a positive ion mode via QToF-MS (Xevo G2-S QToF, Waters MS Technologies, Manchester, UK). For optimization, the capillary voltage and sampling cone voltage were set to 3.5 kV and 40 V, respectively, while the ion source temperature and desolvation temperature were set to 120 and 500 °C, respectively. In addition, the desolvation N_2_ gas and the scan range of the mass were set to 1020 L/h and 100–1200 *m*/*z*, respectively. After the components of individual isoflavone derivatives were analyzed, they were identified by referring to LC-MS fragment ion patterns from the previously prepared soybean library. Quantification of individual isoflavones was performed by calculating a relative quantitative value (mg/100 g, dry weight) by comparing the area of each component 1:1 without considering the relative response factor of the internal standard (6-methoxyflavone) injected during pretreatment [10,11,25].

### 2.6. Statistical Analysis

Data are provided in the format of mean ± standard deviation (SD). The experiment was repeated three times to significantly verify the difference between the measured values. A *t*-test was conducted to determine the significance between the control and experimental groups. Furthermore, Duncan’s post hoc test was applied regarding the difference between samples only when there was a significant difference between groups based on the one-way analysis of variance (ANOVA) [10]. A two-way ANOVA was performed to determine the interaction effects of years on the efficacy and composition of the soybean genetic resources.

For comprehensive understanding of the relationship between the physiological activity of soybean sources and individual isoflavone derivatives, the correlation was determined using principal component analysis (PCA) and a correlation heat map [10].

All statistical analyses were conducted utilizing the Statistical Package for the Social Sciences (SPSS) software (Ver. 18.0, SPSS, Inc., Chicago, IL, USA) and JASP 0.14.1.

### 2.7. DNA Extraction, Genotyping, and Genome-Wide Association Study (GWAS)

A GWAS was conducted to investigate the relationship among the efficacy of soybean sources, the genes related to individual isoflavone derivatives, and molecular regulation mechanisms between traits. Total DNA from 48 soybean seed samples was extracted using the Bead™ Genomic DNA Prep Kit for Plants (Biofact, Daejeon, Korea) according to the manufacturer’s instructions. Total 49 soybean core accessions were genotyped using the 180K AXIOM^®^ SoyaSNP array [26]. Genotyping and quality control procedures were performed according to “Axiom genotyping solution data analysis guide” and “Best practice supplement to Axiom genotyping solution data analysis user guide” (https://assets.thermofisher.com/TFSAssets/LSG/manuals/axiom_genotyping_solution_analysis_guide.pdf, accessed on 10 August 2022). A total of 180,375 SNPs arranged on 20 chromosomes were obtained. Using Tassel 5 to filter out individuals with minor allele frequency below 0.05, missing data on SNP sites above 0.05, and missing data over 0.1, there were 121,453 SNPs remaining, and all 49 individuals passed the filter. On average, approximately 1 SNP is provided per 9.06 kb sequence. Genome-wide association studies were performed using the mixed linear model (MLM) in the GAPIT3 R package [27]. The CMplot R package was used for the combination and visualization of multiple traits of the GWAS results. Bonferroni multiple test thresholds were set at 0.05 and 0.01. To determine the scan range of candidate genes, LD blocks were defined using the confidence intervals algorithm [28] in Haploview v4.2 [29].

## 3. Results

### 3.1. Physiological Activity Analysis of Soybean Seeds

#### 3.1.1. Antioxidant Activity of Soybean Seeds (Radical Scavenging Activity)

Reactive oxygen species (ROS), including hydroxyl radicals, hydrogen peroxide, and superoxide anions, which are overexpressed by oxidative stress in the human body damage DNA, cell membranes, and proteins and enzymes within cells, in addition to inducing other harms, such as cell aging, an increase in cancer cells, and brain damage [30,31]. Thus, the analysis methods of ABTS and DPPH radical scavenging activities are typically utilized to determine how well a food ingredient with high antioxidant activity removes ROS [30,31]. The activity is measured by utilizing the principle in which a radical is reduced due to an antioxidant, resulting in decolorization from its intrinsic color [30,31].

A two-way ANOVA was performed to determine the differences between the cultivation year, seed coat color, and the interaction between the year and seed coat color of the antioxidant activity of soybean seeds (Table 2). As a result, the ABTS and DPPH activities showed no difference by cultivation year, whereas the ABTS radical scavenging activity indicated a difference in seed coat color alone, and the DPPH radical scavenging activity showed differences due to seed coat color and the interaction effects of year and seed coat color.

The ABTS radical scavenging activity increased by approximately 17.8% in the second year compared to the first year [10] in the average values of all samples (Table 2). In the second year, the ABTS activity of all resources ranged from 1.93 to 15.89 mg AA eq/g, with an average of 4.88 mg AA eq/g (Table 3). In terms of color, a higher activity was determined in the order of the black group (6.31 mg AA eq/g) > the yellow group (3.37 mg AA eq/g) > the green group (3.17 mg AA eq/g). The sample with the highest activity in the second year was the black B6 sample (15.89 ± 0.44 mg AA eq/g), which was 2.7 times higher than that of the black control C2 (5.91 ± 0.41 mg AA eq/g). However, B6 was the sample with the greatest difference between the years, showing an increase from 4.11 ± 0.66 mg AA eq/g in the first year to approximately 11.78 mg AA eq/g in the second year. The sample showing the highest activity in the first year was B17 (11.93 ± 1.08 mg AA eq/g), and it retained a high level of activity in the second year (10.93 ± 0.40 mg AA eq/g). The sample showing the smallest difference over the two years was B23, which showed a decrease from 7.98 ± 1.49 mg AA eq/g in the first year to 7.96 ± 0.55 mg AA eq/g in the second year, changing by approximately 0.01 mg AA eq/g [10].

The average value of the DPPH radical scavenging activity in all samples increased by approximately 49.7% in the second year compared to the first year [10] (Table 3). The average value of the DPPH scavenging activity in the second year was 2.77 mg AA eq/g, ranging from 1.02 to 9.23 mg AA eq/g for all samples. In terms of the color of the soybeans, higher activity was determined in the order of the black group (3.63 mg AA eq/g) > the yellow group (1.87 mg AA eq/g) > the green group (1.68 mg AA eq/g), as in the ABTS activity. Similar to the ABTS activity, the highest DPPH activity was found in the B6 sample (9.23 ± 0.49 mg AA eq/g), which had 2.4 times higher activity than the black control, C2 (3.77 ± 0.16 mg AA eq/g). Similar to the ABTS activity, B6 was the sample with the largest year-to-year difference, with its activity increasing from 1.80 ± 0.38 mg AA eq/g in the first year to about 7.43 mg AA eq/g in the second year. The highest activity was found in the B17 sample (6.20 ± 0.35 mg AA eq/g) in the first year, and it retained a high level of activity in the second year (6.27 ± 0.30 mg AA eq/g) [10].

#### 3.1.2. Estrogen and Estrogen Receptor Alpha Activity in MCF-7 Cells

Estrogen, a female hormone, is involved in the important metabolic processes of regulating energy balance, influencing calcium absorption, and regulating immune cell activation and osteoblast activation [32]. As the role of estrogen in vivo is mediated by the estrogen receptor, the expression and activity of this receptor are important factors in which female hormones play a role [32].

A two-way ANOVA was performed to determine the differences in the cultivation year, seed color, and the interaction between the year and seed coat color of the estrogen and estrogen receptor alpha activity of soybean seeds (Table 2). Estrogen receptor alpha showed a difference only in the cultivation year, whereas estrogen showed no difference in any item.

**Figure 1:**
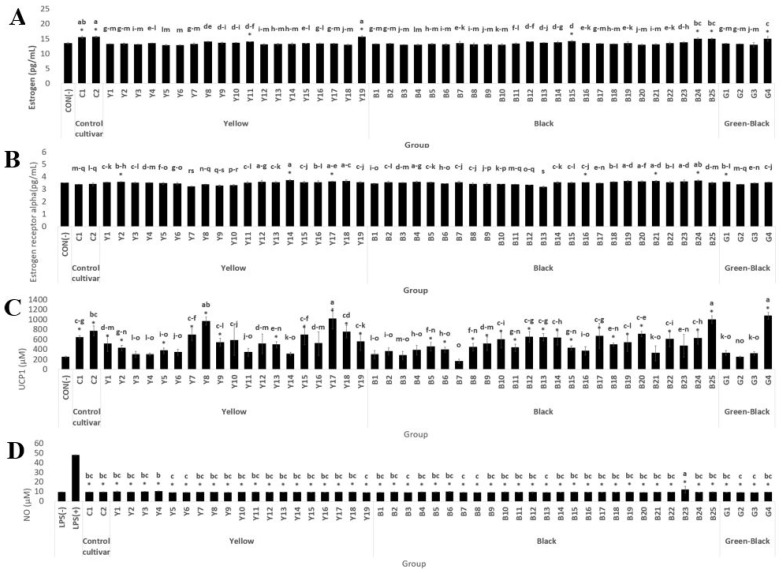
Comparison of (**A**) estrogen activity, (**B**) estrogen receptor alpha activity, (**C**) UCP-1 activity, (**D**) NO inhibition activity by soybean seed coat color. ^a–s^ Means followed by the same letter within rows are significantly different at *p* < 0.05, Duncan’s multiple range test. * Significantly different from CON and LPS (+) at *p* < 0.05.

The estrogen activity analysis showed no significant difference by the cultivation year. Compared to the control (-), the average activity in the second year was 99.99%, and in the first year [10] was 99.96% on average (Table 2). The second-year soybean samples showed a high activity according to color in the order of the green group (13.66 pg/mL) > the black group (13.53 pg/mL) > the yellow group (13.48 pg/mL) (Figure 1A). In the second year, 6 out of the 48 samples had a significantly higher effect than the control (-), whereas in the first year, no sample showed a significant difference compared to the control (-). The samples with significantly higher estrogen activity in the second year were Y11, Y19, B15, B24, B25, and G4.

The average estrogen receptor alpha activity in the second year was 99.91% compared to the control (-), and that activity in the first year [10] was 118.88% compared to the control (-) (Table 2). Soybean samples from the second year showed high activity in the order of the black group (3.52 pg/mL) > the yellow group (3.51 pg/mL) > the green group (3.50 pg/mL) (Figure 1B). The estrogen receptor alpha activity of the first year was significantly increased in about 80% of the samples compared to the control (-), whereas in the second-year samples, only seven resources were significant, with Y2, Y14, Y17, B16, B21, B24, and G1 showing increases. The sample with the highest activity in the second year was Y14, despite its activity being low in the first year, and the sample with the highest activity in the first year was Y10, which showed low activity in the second year [10]. The B24 sample, in contrast, showed high activity in both the first year (9.20 pg/mL) and the second year (3.70 pg/mL) [10].

#### 3.1.3. Antiobesity Activity by Brown Fat Conversion Activity (UCP-1 Activity of C3H10T1/2 Cells)

UCP-1 is present in brown adipose tissue (BAT), which plays a key role in thermoregulation, particularly converting the potential of the membrane into heat in the inner mitochondrial membrane, thereby preventing the energy stored in fat from converting to adenosine triphosphate [33]. UCP-1 in BAT increases energy expenditure through uncoupling oxidative phosphorylation and triggers BAT-mediated thermogenesis, which is activated by the hypothalamus through the sympathetic nerve [34].

A two-way ANOVA was performed to determine the differences in UCP-1 activity of soybean seeds by cultivation year, seed coat color, and the interaction between year and seed coat color (Table 2). There was a difference according to the cultivation year, whereas the activity was unsusceptible to seed coat color and the interaction of year and seed coat color.

According to the UCP-1 analysis, the average activity in the second year was approximately 206.14% compared to the control (-), whereas it was 273.40% in the first year [10]. In the second-year sample, higher brown fat conversion activity was determined in the order of the yellow group (541.67 µM) > the black group (502.65 µM) > the green group (494.40 µM) (Figure 1C). In the first year [10], there was a significant difference in most of the samples compared to the control (-), whereas in the second year, a significant difference was confirmed in approximately 60% of the samples. The sample showing the highest activity in the second year was G4, which had low activity in the first year, whereas the highest sample in the first year was B11, which had low activity in the second year [10]. Y7, in contrast, showed high activity in both the first year (400.13 µM) and second year (695.71 µM) [10].

#### 3.1.4. NO Production Inhibition Activity of RAW 264.7 Cells

NO is produced from L-arginine by nitric oxide synthase (NOS), which is present in various tissues. It plays a key role in various physiological and pathological processes, and it is attracting attention as an inflammatory mediator [35]. However, when overproduction occurs, NO is involved in mutagenesis, DNA damage, altering the function of iron–sulfur-containing enzymes, and disrupting mitochondrial respiration [36].

A two-way ANOVA was performed to determine the differences in the NO production inhibitory activity of soybean seeds according to the cultivation year, seed coat color, and interaction between year and seed coat color (Table 2). There was a difference only in the cultivation year, whereas there were no interaction effects between the seed coat color and year.

Compared with the LPS (+) treatment group, there was a significant difference in the NO production inhibitory activity of all samples in the second year, with an inhibitory effect of approximately 80.44%. In the first year [10], there was a significant difference in approximately 50% of the samples compared to the LPS (+) treatment group, with an average NO production inhibitory effect of 12.94% (Table 2). The NO production inhibitory activity was higher in the second year than in the first year [10]. When the samples in the second year were compared by seed coat color, there was little difference among the yellow, black, and green groups, and no samples showed a high activity in both the first and second years (Figure 1D).

### 3.2. Composition and Content of Isoflavones in Soybean Seed Resources

The individual components of 19 individual isoflavone derivatives (10 types of genistein derivatives, 5 types of daidzein derivatives, and 4 types of glycitein derivatives) of soybean seeds in the second year were observed at UV 254 nm in the same manner as in the first year, and various glycoside forms other than the aglycone structure were identified [11,37]. Moreover, the results of previous studies were confirmed for the composition and content of 19 types of isoflavones, including newly estimated components in addition to 12 key components of soybeans [10,11]. An annual comparison regarding the isoflavone composition and content was performed as a joint study on statistical analysis and comparison based on previously reported research articles [10] and reports [37].

A two-way ANOVA was performed to determine the difference in the isoflavone content of soybean seeds by the cultivation year, seed coat color, and the interaction between year and seed coat color (Table 4). Hereinafter, the 19 individual isoflavone derivatives presented in Table 4 are abbreviated as F1–F19. As a result, the individual derivatives that showed a difference according to the year were F2, F7, F9, and F13–F16, whereas the components that demonstrated a significant difference according to both year and seed coat color were F6, F13, and F15. No derivative showed a difference due to the interaction of year and seed coat color in the seed coat color alone. For the isoflavone groups, there were differences in the daidzein group by year and the genistein group by year and seed coat color. There was a difference in the total isoflavone according to year, whereas there were no effects on the glycitein group.

**Figure 2:**
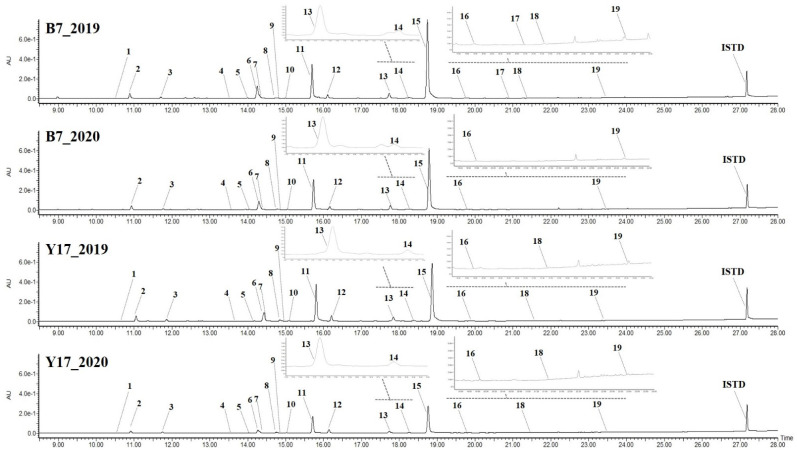
UPLC-DAD chromatogram of 19 isoflavone derivatives (wavelength at 254 nm) from soybean resources B7 and Y17 (year 2019 and 2020). Compound names are presented according to peak numbers in Table 4. ISTD (internal standard): 6-methoxyflavone 50 ppm.

The average of the total isoflavone content of soybean seeds in all samples decreased by approximately 16.58% in the second year compared to the first year [10]. The average of the total isoflavone content of all samples in the second year was 240.07 mg/100 g dry weight (DW), ranging from 69.71 to 472.26 mg/100 g DW. The highest content was found in B7 (472.26 ± 48.64 mg/100 g DW), followed by B1 (432.04 ± 21.48 mg/100 g DW), Y13 (405.83 ± 31.95 mg/100 g DW), Y4 (402.46 ± 27.36 mg/100 g DW), and B18 (350.13 ± 33.57 mg/100 g DW). The content of B7 in the first and second years was 490.56 ± 4.87 mg/100 g DW and 472.26 ± 48.64 mg/100 g DW, respectively, remaining at the highest level over the two-year period. The sample with the greatest difference between the first and second years was Y17, whose content was 387.49 ± 7.21 mg/100 g DW in the first year and 172.66 ± 28.58 mg/100 g DW in the second year, with a decrease of approximately 214.84 mg/100 g DW (Figure 2). The sample with the smallest difference over the two-year period was B9, whose content was 298.01 ± 0.77 mg/100 g DW in the first year and 299.32 ± 26.68 mg/100 g DW in the second year, with a decrease of approximately 1.31 mg/100 g DW [10].

The total content of the daidzein, genistein, and glycitein groups in the soybean seeds decreased by 16.01, 19.18, and 9.18% in the second year, respectively [11], compared to the first year [10]. The total content of the daidzein group of soybean seeds in the second year ranged from 26.07 to 182.01 mg/100 g DW, and the average of the samples was 81.69 mg/100 g DW [11]. The total content of the genistein group ranged from 37.48 to 322.24 mg/100 g DW, and the average of the samples was 139.24 mg/100 g DW. The total content of the glycitein group ranged from 0.00 to 41.59 mg/100 g DW, and the average of the samples was 14.53 mg/100 g DW.

### 3.3. PCA and Correlation Analysis between Efficacy and Components of Soybean Samples

**Figure 3:**
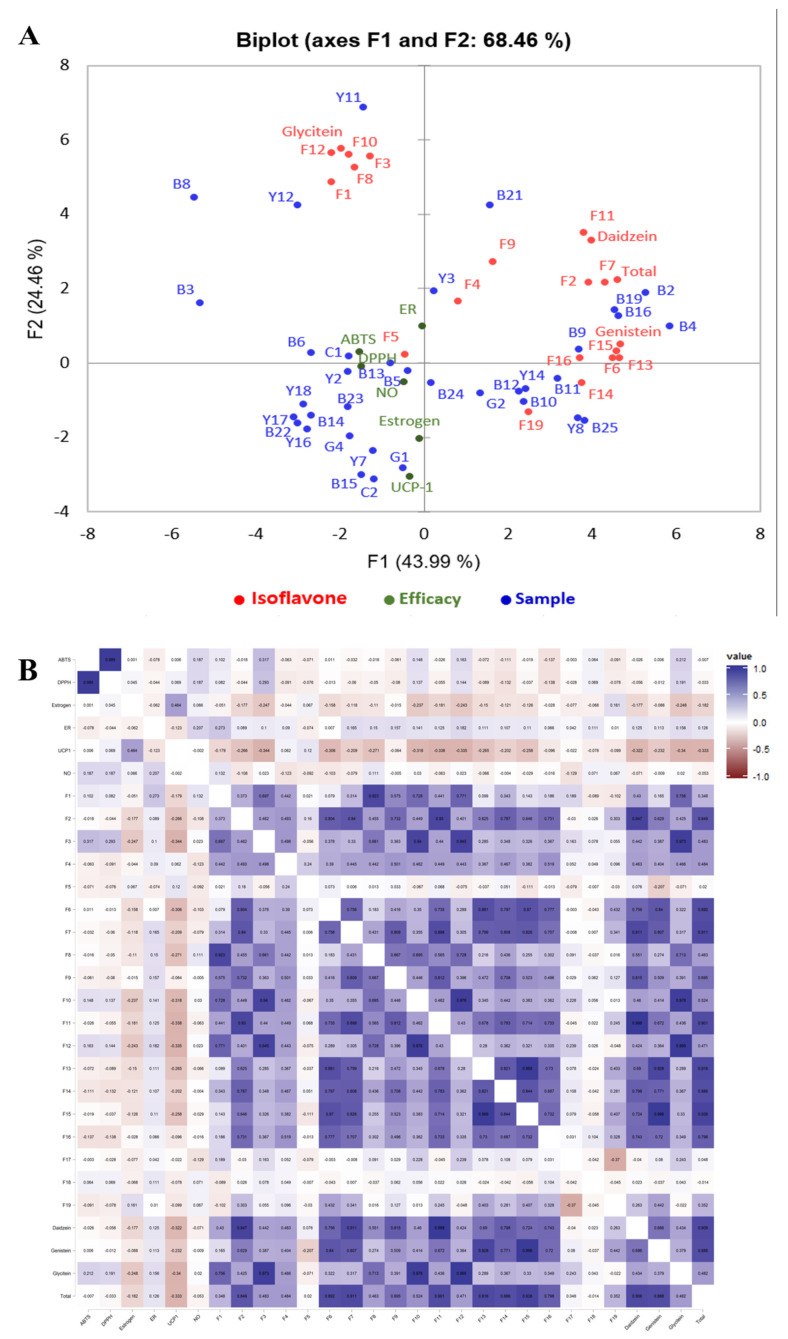
(**A**) Analysis of isoflavones, effects and samples by PCA. ABTS, radical scavenging activity; DPPH, radical scavenging activity; Estrogen, estrogen activity; ER−α, estrogen receptor alpha activity; UCP−1, uncoupling protein−1 activity; NO, nitric oxide inhibition activity. Control cultivar, C1−C2; Yellow group, Y1−Y19; Black group, B1−B25; Green group, G1−G4; Daidzein, daidzein group; Genistein, genistein group; Glycitein, glycitein group; Total, total isoflavone. (**B**) Correlation analysis of effects and isoflavone with soybean seed. F1, genistein 5-*O*-glucoside; F2, daidzein 7-*O*-glucoside (daidzin); F3, glycitein 7-*O*-glucoside (glycitin); F4, genistein 7-*O*-(6″-*O*-apiosyl)glucoside; F5, genistein 7-*O*-(2″-*O*-apiosyl)glucoside; F6, genistein 7-*O*-glucoside (genistin); F7, daidzein 4′-*O*-(6″-*O*-malonyl)glucoside; F8, genistein 5-*O*-(6″-*O*-malonyl)glucoside; F9, daidzein 7-*O*-(4″-*O*-malonyl)glucoside (4″-*O*-malonyldaidzin); F10, glycitein 4′-*O*-(6″-*O*-malonyl)glucoside; F11, daidzein 7-*O*-(6″-*O*-malonyl)glucoside (6″-*O*-malonyldaidzin); F12, glycitein 7-*O*-(6″-*O*-malonyl)glucoside (6″-*O*-malonylglycitin); F13, genistein 4′-*O*-(6″-*O*-malonyl)glucoside; F14, genistein 7-*O*-(4″-*O*-malonyl)glucoside (4″-*O*-malonylgenistin); F15, genistein 7-*O*-(6″-*O*-malonyl)glucoside (6″-*O*-malonylgenistin); F16, daidzein; F17, glycitein; F18, genistein 7-*O*-(6″-*O*-acetyl)glucoside (6″-*O*-acetylgenistin); F19, genistein; Daidzein, daidzein group; Genistein, genistein group; Glycitein, glycitein group; Total, total isoflavone.

PCA was conducted to comprehensively analyze the relationship among 19 individual isoflavone derivative components, 6 types of efficacy, and 48 types of soybean sources (50 types, including control cultivar samples), and this analysis was conducted based on the obtained results, excluding the samples with a low correlation (Figure 3A). The first principal component (PC1) and the second principal component (PC2) explained 43.99 and 24.46% of the variation, respectively, amounting to 68.46%. For PC1, estrogen and UCP-1 were loaded in the negative (−) direction and were close to each other, and ABTS and DPPH radical scavenging activities, NO production inhibitory activity, and estrogen receptor alpha were close to each other and loaded in the negative (−) direction. In contrast, genistein 7-*O*-(2″-*O*-apiosyl)glucoside (F5) was determined to be the individual isoflavone derivative located closest to this efficacy. According to the relationship between samples and efficacy, estrogen and UCP-1 were located close to G1, a green-black group, and B5 and B13 in the black group were located close to NO and estrogen receptor alpha. The ABTS and DPPH (antioxidant activities) were located close to C1 and Y2, suggesting a correlation between them. The substances located in the negative (−) positions, which are the same positions as the efficacy based on PC1, were F1, F3, F8, F10, F12, and glycitein.

F2, F7, and F11 of the daidzein family were located close to the daidzein group, and the individual components F6, F13, F15, and F16 of the genistein family were located close to the genistein group. The individual components of the glycitein group, F3, F8, F10, and F12, were located close to the glycitein group. Total isoflavone was located in the same main component (PC1) as the genistein and daidzein groups, revealing a closer relationship between them.

In the first year, the components with a high correlation with six types of efficacy were F5, F17, and F18, and the correlation was confirmed for these components in the second year, preventing statistical treatment due to the trace amounts of F17 and F18 (0 value comprised 85% of the samples) [10]. Accordingly, a re-analysis excluding F17 and F18 showed that F5 was the most correlated component, regardless of year (Figure 3B).

Pearson’s correlation coefficients were calculated to examine the correlations between the six types of physiological activity of the soybean samples and individual isoflavone derivative components, which were presented as a heat map (Figure 2). In the relationship between efficacy and efficacy, ABTS and DPPH radical scavenging activities showed a high correlation, with a correlation coefficient of 0.989, and estrogen and UCP-1 showed a correlation, with a correlation coefficient of 0.464.

**Figure 4:**
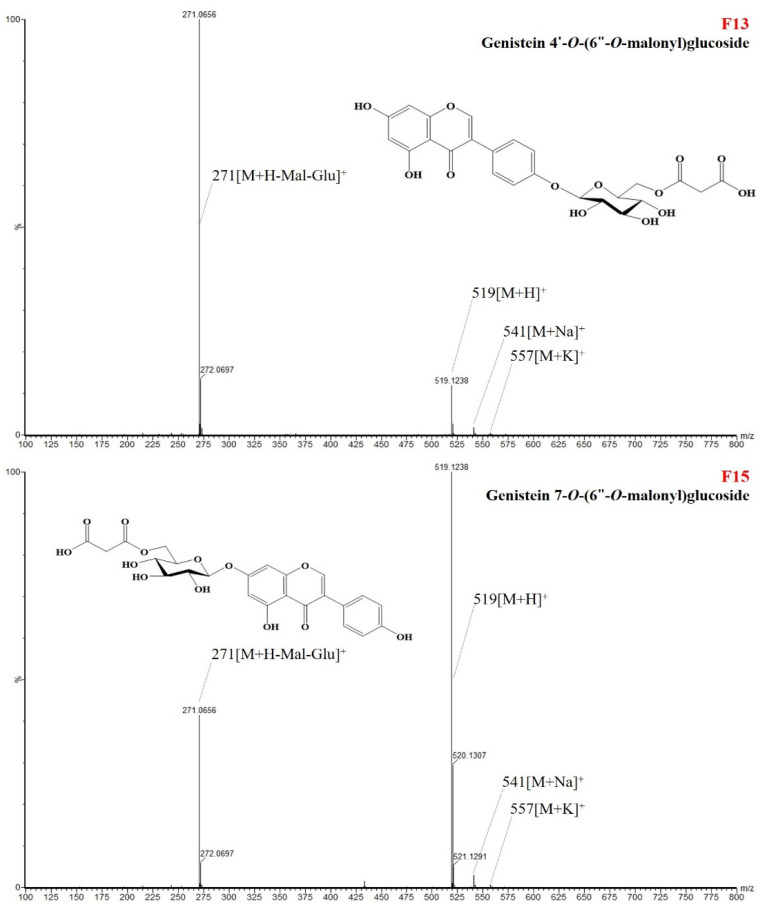
Structures and ESI (+)-QToF/MS fragmentations (*m*/*z*, 519 [M + H]^+^) of two representative isoflavone derivatives (F13 and F15) in soybean (*Glycine max* L.) seeds.

The correlation between the 19 individual isoflavone derivatives was mostly determined to be positive. Genistein 4′-*O*-(6″-*O*-malonyl)glucoside (F13) and genistein 7-*O*-(6″-*O*-malonyl)glucoside (6″-*O*-malonylgenistin) (F15) showed the highest correlation, with a correlation coefficient of 0.989 (Figure 4). Glycitein 4′-*O*-(6″-*O*-malonyl)glucoside (F10) and glycitein 7-*O*-(6″-*O*-malonyl)glucoside (6″-*O*-malonylglycitin) (F12) also demonstrated a high correlation, with a correlation coefficient of 0.976. Both of these components showed a high positive correlation with glycitein 7-O glucoside (glycitin) (F3), with correlation coefficients of 0.943 and 0.947, respectively.

The correlation between the three isoflavone groups, total isoflavone, and individual derivatives of the soybean samples revealed that the correlation coefficients of F2, F7, and F11 of the daidzein group were 0.947, 0.911, and 0.998, respectively, indicating a high correlation with the daidzein group. The correlation coefficients of F13 and F15 of the genistein group were determined to be 0.928 and 0.996, respectively, indicating a high correlation with the genistein group. The correlation coefficients of F3, F10, and F12 of the glycitein group were determined to be 0.973, 0.978, and 0.995, respectively, showing a high correlation with the glycitein group. The correlation coefficients of total isoflavone were determined to be 0.916 and 0.938, respectively, indicating a close relationship with the genistein group (F13 and F15).

The investigation of the correlation between the physiological activity and individual isoflavone derivatives revealed a low correlation coefficient. However, the samples with a correlation coefficient of ±0.3 or more were glycitein 7-*O*-glucoside (glycitin) (F3) and ABTS, which demonstrated a positive (+) correlation with a correlation coefficient of 0.317, while F3, F6, F10, F11, and F12 showed negative (−) correlations.

### 3.4. Understanding the Relationship between Individual Isoflavone Derivatives and the High Efficacy of Soybean Resources and Genetic Properties

**Figure 5:**
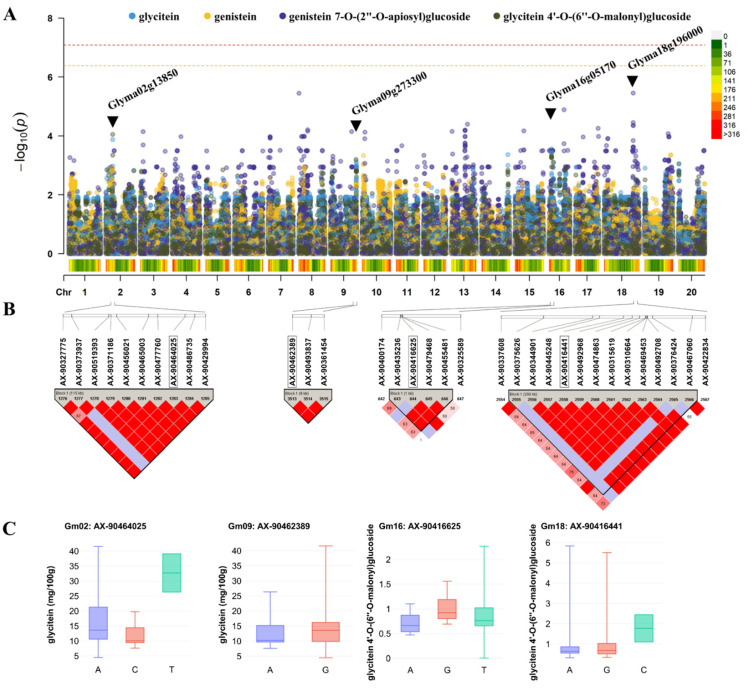
Four flavonoid-related genes identified by GWAS. (**A**) Four QTLs derived from GWAS. (**B**) LD block used to determine the range of candidate genes. (**C**) Boxplots showing differences in flavonoid content across alleles.

A GWAS was conducted to investigate the relationship between the genetic properties of individual isoflavone derivatives that were highly correlated and the efficacy of soybean sources. A GWAS is typically utilized to identify the location of genes associated with a trait of interest in a crop by exploring the single nucleotide polymorphism (SNP) of a crop, thereby determining the association between the genetic markers and natural population traits [10]. Based on the PCA result, a GWAS was conducted regarding the individual isoflavone derivatives F1, F3, F8, F10, and F12 and the daidzein, genistein, and glycitein groups and total isoflavone, including F5 located in the same direction (considered to be related), with the six types of physiological activity based on PC1 (43.99%) and total isoflavone. There was no genetic association between the six types of physiological activity and these components. Among these derivatives, this study presents the results of the four components of the glycitein and genistein groups from which the GWAS results were derived, as well as genistein 7-*O*-(2″-*O*-apiosyl)glucoside (F5) and glycitein 4′-*O*-(6″-*O*-malonyl)glucoside (F10) (Figure 5).

Therefore, the gene involved in the generation of each of the four components was examined in detail. The GWAS results showed that both glycitein and glycitein 4′-*O*-(6″-*O*-malonyl)glucoside were associated with AX-90464025 on chromosome 2, AX-90462389 on chromosome 9, and AX-90416625 on chromosome 16. Genistein 7-*O*-(2″-*O*-apiosyl)glucoside was associated with AX-90416441 on chromosome 18. The range of the LD blocks derived from the Haplotype analysis was scanned on the reference genome Glyma1 [38], published by SoyBase (https://www.soybase.org/, accessed on 11 August 2022). In the LD block where AX-90464025 on chromosome 2 was located, the most likely candidate gene is Glyma02g13850, as a probable 2-oxoglutarate/Fe (II)-dependent dioxygenase-like [Glycine max]. According to the given ontological interpretation in AmiGO 2 (http://amigo.geneontology.org/amigo/term/GO:0016491/, accessed on 11 August 2022), it is shown that this gene may be associated with flavonol synthase in soybeans. The GO class (direct) is flavonol synthase activity, dioxygenase activity, and response to light stimulus. No studies have shown that this gene regulates glycitein 4′-*O*-(6″-*O*-malonyl)glucoside and glycitein content.

In the LD block where AX-90462389 on chromosome 9 was located, the most likely candidate gene is Glyma.09g273300 (FERONIA receptor-like kinase). In a previous study, FERONIA-like receptor kinase FaMRLK47 mainly played a role in the regulation of anthocyanin accumulation, flavonoid metabolism, and fruit softness [39]. As an upstream regulatory gene, FaMRLK47 is responsible for regulating the expression of FaCHS, FaCHI, FaUFGT, FaPAL, FaPE, FaPG, FaXYL1, and FaEXP1. Furthermore, FaMRLK47 mediates ABA production and signal transduction. The LD block of AX-90416625 on chromosome 16 was only 1kb, and the only one candidate gene is Glyma16g05170 (receptor-like protein kinase 2). In rice, receptor-like kinase can phosphorylate a basic leucine zipper (bZIP) transcription factor [40]. The bZIP binding to the promoters of CHS, CYP93G2, OsbZIP48, 4CL5, and C4H or enhancing the activity of proOsbZIP48:LUC positively regulates the flavonoid metabolism.

In the LD block where AX-90416441 on chromosome 18 was located, the most likely candidate gene is Glyma18g196000 (acetyl-CoA carboxylase). Previous studies have shown that acetyl-CoA carboxylase catalyzes the first two steps of flavonoid and fatty acid biosynthesis pathways in plants [40,41].

In addition, there are other QTLs associated with the target trait. However, the candidate genes in these QTLs may be related to light response rather than flavonoid synthesis-related genes. For example, in the LD block where AX-90462389 of chromosome 20 was located, there are three candidate genes, and no upstream gene for flavonoid synthesis was found. Regarding Glyma.20G012000 (nuclear ribonuclease Z), no study mentions the relationship between this gene and flavonoids. With regards to Glyma20g01490 (a hydroxyproline-rich glycoprotein family protein), hydroxyproline-rich glycoprotein (HRGP) is a major factor in cell wall strengthening. With regards to Glyma20g01505 (anthranilate synthase), UVB may lead to a significant enhancement in flavonoids and tryptophan, with a concomitant decrease in auxin signaling [42]. However, anthranilate synthase, which encodes the main enzyme of the indole/tryptophan pathway, is not a causal gene for flavonoid synthesis. In the following research, we will study the expression and gene function verification of these genes that may be related to flavonoid synthesis in order to clarify the soy flavonoid synthesis pathway.

## 4. Discussion

Forty-eight soybean seeds were sown and harvested at the same time of year and place during a two-year study period to investigate the change in the relationship between the physiological activity and content of individual isoflavone derivatives, and their efficacy, substances, and genetic properties.

First, the items whose efficacy increased more in the second year than in the first year showed ABTS and DPPH radical scavenging activities and NO production inhibitory activity, among the six types of physiological activity, as well as genistein 5-*O*-(6″-*O*-malonyl)glucoside (F8) among 19 individual isoflavone derivatives.

According to the results of the two-way ANOVA analysis (yearly and seed coat color) of the ABTS and DPPH radical scavenging activities, which are the antioxidant activities of soybeans, there was a significant difference in the seed coat color for the ABTS radical scavenging activity, as well as an interaction between year and seed coat color for the DPPH radical scavenging activity. The ABTS showed no difference by year, showing a high environmental stability and no effect of the color of the seed coat due to genetic properties. Among the seed coat colors, the black group showed the highest activity in both the first and second years [10]. Some studies have reported that the anthocyanin-based component of the seed coat color contributes to increasing the antioxidant activity of the soybeans and that the antioxidant activity decreases as the color of the seed coat of soybeans becomes lighter [43,44]. The samples that maintained the highest activity in both the first and second years were B17, B15, and B23 in the black group, revealing that the traits remained stable in these samples despite climatic variations [22].

The NO production inhibitory activity of soybeans increased in the second year, and unlike the antioxidant activity, it showed a significant difference in the ANOVA results between the years compared to the seed coat color, which could be attributed to the effect of the cultivation environment (Table 3). Jeong et al. [8] showed that the NO production inhibitory activity becomes higher as the ABTS and DPPH radical scavenging activities of the sample group treated with bean pod extracts increases, which was confirmed in this study, suggesting that the anti-inflammatory effect increases as the antioxidant activity becomes higher. Moreover, among the isoflavones of soybeans, genistein showed a high anti-inflammatory effect, and it has been found that aglycone is more effective at inhibiting NO production than glycoside [45]. As no samples stably maintained high NO production inhibitory activity in both years, this property was determined to have low environmental stability, varying depending on the cultivation characteristics.

Although the content of both total isoflavone and individual isoflavone derivatives mostly decreased, plants have been reported to contain more antioxidants when the cultivation environment is poor based on the increase in antioxidant and anti-inflammatory properties [46,47]; thus, these decreases could be attributed to the deterioration of the cultivation environment in the second year. In addition, flavonoid-based anthocyanins, known as antioxidants, have been reported to increase under shade stress conditions [48]. Soybean crops that are exposed to shade develop a mechanism of converting carbon to produce secondary metabolites, such as anthocyanins and proanthocyanidins, to overcome shade stress [48]. In 2020, the rain was concentrated in July and August, corresponding to the flowering season, and the duration of daylight decreased accordingly. The sum of the duration of daylight from June to September was 620.7 h in the normal year, whereas it amounted to 672.5 h in 2019, which was 51.8 h longer than in the normal year. The sum was 585.3 h in 2020, which was 35.4 h shorter than the normal year [22]. The decrease in the duration of daylight would have had the same effect as the shade stress, resulting in increased antioxidant components and efficacy due to the increase in anthocyanins.

In the annual comparison of the physiological activity and isoflavone derivatives which decreased in the second year rather than in the first year, estrogen, estrogen receptor alpha, UCP-1 efficacy, 19 individual derivatives, 3 isoflavone groups and total isoflavone are included. There was no significant difference in estrogen in the ANOVA results between the years and the seed coat colors, while there was a significant difference in the estrogen receptor alpha only between the years (Table 2). As the isoflavones of soybeans exhibit similar activity to estrogen [49], a decrease in the isoflavone content results in a reduction in the activity of estrogen and estrogen receptor alpha with respect to the MCF-7 cells. As shown in this study, the isoflavone content and the activity of estrogen and estrogen receptor alpha in soybeans decreased in the second year, which could be attributed mainly to climatic variation. The precipitation from mid-July to early August, which corresponded to the flowering season of the soybean genetic resources, was 196 mm in 2019 (the first year) and 930 mm in 2020 (the second year), which was much higher than the normal annual precipitation of 274 mm. This result suggests that the content of isoflavones decreased due to high waterlogging conditions and low sunlight [22]. Isoflavones, as a secondary metabolite, showed differences in the content and composition ratio according to environmental changes, such as temperature difference and disease occurrence, which is consistent with the previous studies [22]. There was no sample in which estrogen had high activity for both years and maintained it at a stable level, whereas the B24 sample showed a high activity of estrogen receptor alpha and stable efficacy for both years.

According to the ANOVA result between years and seed coat colors, there was a significant difference in the UCP-1 of soybeans according to the year, as their activity decreased more in the second year than in the first year. UCP-1 activity is a brown fat conversion activity, which is one of the indicators related to fat metabolism and obesity [8]. Year-to-year differences in the UCP-1 efficacy could be attributed to the change in the isoflavone content due to climatic variations during the two-year period of soybean cultivation, as well as differences in the fatty acid content and composition [22]. In particular, the genistein in soybeans activates Sirt1 and promotes the expression of UCP-1, thereby stimulating the conversion of white fat into brown fat [50]. Thus, as previously described, a decrease in genistein in the isoflavone content results in the reduced activity of UCP-1. Furthermore, oleic acid, which is one of the essential fatty acids in soybeans, reduces weight gain and body fat accumulation, which is highly related to thermogenesis control and an improvement in the lipid metabolism in liver and skeletal muscle [51,52]. Studies have reported that the concentration of oleic acid is greatly affected by the year rather than the cultivation area [53,54] and that the concentration increases depend particularly on environmental factors, such as high temperatures and drought [55]. The decrease in the antiobesity effect of soybean samples could be attributed to a decrease in fatty acids rich in soybeans or a change in their composition because there was waterlogging in 2020 (the second year) due to high precipitation compared to 2019 (the first year). The Y7 sample (IT195514) in this study showed a high UCP-1 activity in the first and second years, which supports our prediction because this sample was reported to have a high total oil content and a higher oleic acid content than the other samples with yellow-colored seed coats [35].

Second, according to the PCA on the relationship between the efficacy, substances, and genetic properties, three components (F5, F17, and F18) in 2019 and one component (F5) in 2020 were determined to be highly related to physiological activity. Although three individual substances (F5, F17, and F18) were present in 2020 and were the same as those in 2019, based on the first statistical analysis result, the decrease in the isoflavone content of the samples grown in the second year resulted in a low content of the individual isoflavone derivatives. Due to the trace content of F17 and F18 in the soybean resources and that the 0 value comprised approximately 85% of the total samples (data not shown), making them statistically insufficient as data, an additional statistical analysis was conducted, excluding F17 and F18. The genistein 7-*O*-(2″-*O*-apiosyl)glucoside (F5), an individual isoflavone derivative, was predicted to be a substance with the highest efficacy and correlation in both the first and second years, regardless of the cultivation environment. F5 was the first substance identified in soybeans by the other research team conducting joint research for this study [11]. This trace amount of substance contained in soybeans will affect various physiological activities as a member of the genistein group [25,56]. Several studies have reported the beneficial health effects of isoflavone genistein, such as alleviating menopausal symptoms and reducing the incidence of cardiovascular diseases, osteoporosis, obesity, diabetes, and cognitive impairment [57].

According to the correlation analysis result using a heat map, the correlations between physiological activity, individual isoflavone derivatives, and components and efficacy were lower in 2020 than in 2019. This is because, as previously described, the environmental impact of open-field cultivation was dominant despite the varieties, cultivation soil, and sowing and cultivation period being identical [22]. As a result, the quantity-related traits decreased, such as the number of seeds per pod, the number of pods per plant, and 100-seed weight [22], and there was a low significant correlation between the physiological activity and the component content in this sample. ABTS-DPPH (2019, 0.835; 2020, 0.989) showed the same results for both years, with a high correlation coefficient between the physiological activities, and four substances displayed the same results for both years, with a high correlation coefficient between the individual isoflavone derivatives: F1–F8 (2019, 0.935; 2020, 0.923), F3–F12 (2019, 0.934; 2020, 0.945), F10–F12 (2019, 0.959; 2020, 0.976), and F13–F15 (2019, 0.975; 2020, 0.989). A correlation greater than ±0.3 in composition and efficacy was observed in ABTS and some of the individual isoflavone derivatives. The correlation between components and efficacy was observed in different substances in the first and second years. One component with a high correlation with efficacy could not be specified in this context, and the relationship varied depending on the cultivation environment.

The PCA applied the GWAS to understand the genetic association of six types of individual isoflavone derivatives, three isoflavone groups, and total isoflavone that had shown a high correlation with the efficacy of soybean resources, offering no meaningful results. In the first year, all six types of physiological activity were determined to be highly correlated with the genetic properties of the three selected individual derivatives [10], whereas in the second year, a genetic association between efficacy and individual derivatives was not found. Most of the soybean resources in the second year exhibited insufficient functional characteristics in most samples due to the overall poor crop yield due to drastic climatic variations [22], which prevented the derivation of a certain relationship between statistical analysis and genetic properties.

## 5. Conclusions

This study, based on ABTS, DPPH, estrogen receptor alpha, and UCP-1, presents B17, B23, B15, B24, and Y7 as samples with a high efficacy and cultivation stability. As described in the Results Section, samples with different efficacies may be selected depending on the intended functions. This study provides the results of joint research regarding efficacy, components, and genetic properties, as well as an analysis of the agricultural traits and cultivation characteristics by directly sowing and cultivating a large number of samples (48 varieties) for two years at the Rural Development Administration in South Korea. The results of this study are significant in that they present a comprehensive description by applying various statistical techniques to analyze the results on efficacy, components, and genetic properties. Despite the annual composition of two years, this study was able to identify a significant change in the efficacy and components of soybeans due to climatic variations; therefore, long-term studies are needed in the future.

## Figures and Tables

**Table 1:** Classification by different seed coat colors and information on soybean seeds (Reprinted with permission from Ref. [10]. 2021, Chu et al.).

Seed Coat Color	Code	Introduction Number	Name	Origin	Cultivar Type
Control	C1 (Yellow)	IT 212859	Daewon	Korea	Control cultivar
	C2 (Black)	IT 213192	Cheongja 2	Korea	Control cultivar
Yellow	Y1	IT 024099	YJ208-1	Korea	Landrace
	Y2	IT 104690	Kongnamul Kong	Korea	Landrace
	Y3	IT 113218	Kongnamul Kong	Korea	Landrace
	Y4	IT 153844	KLS 87248	Korea	Landrace
	Y5	IT 155963	Nongrim 51	Japan	Variety
	Y6	IT 171080	PI 467319	China	Variety
	Y7	IT 195514	Jang Kong	Korea	Landrace
	Y8	IT 219581	Myungjunamul Kong	Korea	Variety
	Y9	IT 229418	Danmi 2	Korea	Variety
	Y10	IT 229421	Hoseo	Korea	Variety
	Y11	IT 231360	Kongnamul Kong	Korea	Landrace
	Y12	IT 263155	Sinpaldalkong 2	Korea	Variety
	Y13	IT 263167	Uram	Korea	Variety
	Y14	IT 263852	Chungnamyeongi-1997-3	Korea	Landrace
	Y15	IT 269982	Milyang 247	Korea	Breeding line
	Y16	IT 270002	Jungmo 3008	Korea	Variety
	Y17	IT 274571	GNU-2007-14613	Korea	Landrace
	Y18	IT 274592	GNU-2007-14723	Korea	Landrace
	Y19	IT 324099	CS 00728	China	Breeding line
Black	B1	IT 021665	PI 90763	China	Landrace
	B2	IT 143347	KLS86185	Korea	Landrace
	B3	IT 161904	PI 84578	Korea	Landrace
	B4	IT 177271	Geomjeong Kong-5	Korea	Landrace
	B5	IT 177573	Geomjeong Kong-5	Korea	Landrace
	B6	IT 177709	Geomjeong Kong-4	Korea	Landrace
	B7	IT 178054	Geomjeong Kong-1	Korea	Landrace
	B8	IT 186183	Kongnamul Kong	Korea	Landrace
	B9	IT 189215	94yuja4	Korea	Landrace
	B10	IT 194560	Geomjeong Kong	Korea	Landrace
	B11	IT 212805	Chungnamseocheon-1999-98	Korea	Landrace
	B12	IT 224192	Jeonbukgunsansujip	Korea	Landrace
	B13	IT 228822	409	Korea	Landrace
	B14	IT 231544	Jwinuni Kong	Korea	Landrace
	B15	IT 239896	Jwineori Kong	Korea	Landrace
	B16	IT 252252	Neoljeokseoritae	Korea	Landrace
	B17	IT 252748	294	Korea	Landrace
	B18	IT 252768	326	Korea	Landrace
	B19	IT 263853	Geomen Kong	Korea	Landrace
	B20	IT 274515	GNU-2007-14502	Korea	Landrace
	B21	IT 275005	197	Korea	Landrace
	B22	IT 308619	Junyeori Kong	Korea	Landrace
	B23	IT 311261	Jwinuni Kong	Korea	Landrace
	B24	K 137773	Heugseong	Korea	Variety
	B25	IT 194558	Geomjeong Kong	Korea	Landrace
Green-Black	G1	IT 154351	KAS579-1	Korea	Landrace
	G2	IT 154724	KAS651-21	Korea	Landrace
	G3	IT 178160	Geomjeong Kong	Korea	Landrace
	G4	IT 186048	Gangwonyanggu-1994-3709	Korea	Landrace

**Table 2:** ANOVA for the six effects of 48 soybean germplasm in 2019 and 2020.

Seed Coat Color	Effects (Unit)	Year	Mean	S.D.	*p* Value		
					Year	Seed Coat Color	Year and Seed Coat Color ^(1)^
Yellow, Black, Green- Black	ABTS ^(2)^ (mg AA eq/g)	2019	4.14	2.00	0.435	<0.001	0.206
		2020	4.88	2.81			
	DPPH ^(2)^ (mg AA eq/g)	2019	1.85	1.06	0.069	<0.001	0.038
		2020	2.77	1.70			
	Estrogen ^(2)^ (%, vs. Control)	2019	99.96	2.75	0.998	0.980	0.776
		2020	99.59	4.47			
	ER ^(2)^ (%, vs. Control)	2019	118.88	12.05	<0.001	0.339	0.336
		2020	99.91	3.29			
	UCP-1 ^(2)^ (%, vs. Control)	2019	273.40	91.25	0.002	0.821	0.346
		2020	206.14	82.3			
	NO ^(2)^ (%, vs. Control)	2019	12.94	14.87	<0.001	0.552	0.473
		2020	80.44	0.96			

^(1)^ *p* value by two-way ANOVA. ^(2)^ ABTS, ABTS radical scavenging activity; DPPH, DPPH radical scavenging activity; Estrogen, estrogen-like activity; ER, estrogen receptor alpha activity; UCP-1, UCP-1 activity; NO, NO production inhibition activity.

**Table 3:** ABTS and DPPH radical scavenging activities in soybean seeds.

Seed Coat Color	Code	ABTS (mg AA eq/g)	DPPH (mg AA eq/g)
Control cultivar	C1	4.45 ± 0.24 ^l,m^	2.68 ± 0.21 ^l–n^
	C2	5.91 ± 0.41 ^h^	3.77 ± 0.16 ^gh^
	Range	4.45–5.91	2.68–3.77
	Mean	5.18	3.23
Yellow	Y1	3.86 ± 0.23 ^n–q^	2.48 ± 0.41 ^m–p^
	Y2	2.08 ± 0.13 ^z^	1.35 ± 0.15 ^v–x^
	Y3	2.26 ± 0.13 ^yz^	1.04 ± 0.10 ^x^
	Y4	3.93 ± 0.20 ^n–p^	1.99 ± 0.19 ^q–t^
	Y5	3.22 ± 0.11 ^s–w^	1.74 ± 0.03 ^s–v^
	Y6	3.22 ± 0.08 ^s–w^	1.64 ± 0.14 ^t–w^
	Y7	3.78 ± 0.14 ^n–r^	1.83 ± 0.12 ^r–v^
	Y8	3.39 ± 0.13 ^q–u^	1.83 ± 0.11 ^r–v^
	Y9	3.74 ± 0.17 ^n–r^	2.06 ± 0.12 ^p–t^
	Y10	3.88 ± 0.17 ^n–q^	2.38 ± 0.13 ^m–q^
	Y11	3.47 ± 0.13 ^p–u^	1.45 ± 0.13 ^u–x^
	Y12	4.13 ± 0.03 ^mn^	1.90 ± 0.19 ^r–u^
	Y13	3.97 ± 0.35 ^no^	1.89 ± 0.17 ^r–u^
	Y14	1.98 ± 0.04 ^z^	1.02 ± 0.10 ^x^
	Y15	1.93 ± 0.19 ^z^	1.45 ± 0.02 ^u–x^
	Y16	5.49 ± 0.39 ^hi^	3.00 ± 0.10 ^j–l^
	Y17	3.65 ± 0.17 ^n–s^	2.38 ± 0.12 ^m–q^
	Y18	3.05 ± 0.10 ^u–x^	2.22 ± 0.06 ^n–r^
	Y19	3.08 ± 0.08 ^t–w^	1.93 ± 0.19 ^q–u^
	Range	1.93–5.91	1.02–3.77
	Mean	3.37	1.87
Black	B1	10.40 ± 0.43 ^d^	6.31 ± 0.27 ^c^
	B2	2.81 ± 0.17 ^wx^	1.36 ± 0.09 ^v–x^
	B3	3.53 ± 0.08 ^o–u^	1.69 ± 0.13 ^s–w^
	B4	4.69 ± 0.12 ^j–l^	2.48 ± 0.38 ^m–p^
	B5	4.76 ± 0.21 ^j–l^	2.66 ± 0.39 ^l–n^
	B6	15.89 ± 0.44 ^a^	9.23 ± 0.49 ^a^
	B7	3.44 ± 0.23 ^p–u^	1.94 ± 0.25 ^q–t^
	B8	12.60 ± 0.50 ^b^	7.41 ± 0.26 ^b^
	B9	5.01 ± 0.31 ^i–k^	2.77 ± 0.56 ^l,m^
	B10	5.54 ± 0.13 ^h^	3.54 ± 0.40 ^hi^
	B11	3.70 ± 0.19 ^n–s^	1.79 ± 0.08 ^r–v^
	B12	3.30 ± 0.16 ^r–v^	1.61 ± 0.04 ^t–w^
	B13	5.10 ± 0.09 ^ij^	2.83 ± 0.05 ^k–m^
	B14	7.59 ± 0.20 ^f^	4.10 ± 0.18 ^fg^
	B15	8.72 ± 0.34 ^e^	4.97 ± 0.21 ^d^
	B16	3.44 ± 0.24 ^p–u^	1.81 ± 0.28 ^r–v^
	B17	10.93 ± 0.40 ^c^	6.27 ± 0.30 ^c^
	B18	4.57 ± 0.20 ^k–m^	2.70 ± 0.22 ^l,m^
	B19	6.55 ± 0.12 ^g^	3.80 ± 0.15 ^gh^
	B20	4.13 ± 0.18 ^mn^	2.57 ± 0.13 ^l–o^
	B21	5.65 ± 0.26 ^h^	3.37 ± 0.54 ^ij^
	B22	5.77 ± 0.10 ^h^	3.44 ± 0.28 ^g–i^
	B23	7.96 ± 0.55 ^f^	4.59 ± 0.31 ^de^
	B24	4.92 ± 0.59 ^i–k^	3.22 ± 0.32 ^i–k^
	B25	6.64 ± 0.33 ^g^	4.39 ± 0.32 ^ef^
	Range	2.81–15.89	1.36–9.23
	Mean	6.31	3.63
Green-Black	G1	3.67 ± 0.18 ^n–s^	2.17 ± 0.13 ^o–s^
	G2	2.88 ± 0.22 ^v–x^	1.24 ± 0.04 ^wx^
	G3	2.61 ± 0.16 ^xy^	1.40 ± 0.35 ^v–x^
	G4	3.55 ± 0.20 ^o–t^	1.90 ± 0.29 ^r–u^
	Range	2.61–3.67	1.24–2.17
	Mean	3.17	1.68

^a–z^ Means followed by the same letter within rows are significantly different at *p* < 0.05, Duncan’s multiple range test.

**Table 4:** ANOVA for the 19 individual isoflavone derivatives and four groups of 48 soybean germplasm in 2019 and 2020.

Seed Coat Color	Components	Year	Mean (mg/100 g)	S.D. (mg/100 g)	*p* Value		
					Year	Seed Coat Color	Year and Seed Coat Color ^(1)^
Yellow, Black, Green- Black	Genistein 5-*O*-glucoside (F1)	2019	0.36	0.35	0.291	0.379	0.978
		2020	0.27	0.28			
	Daidzein 7-*O*-glucoside (daidzin) (F2)	2019	12.79	6.35	0.003	0.637	0.259
		2020	9.32	4.47			
	Glycitein 7-*O*-glucoside (glycitin) (F3)	2019	4.67	2.84	0.772	0.166	0.362
		2020	4.47	2.57			
	Genistein 7-*O*-(6″-*O*-apiosyl)glucoside (ambosin) (F4)	2019	0.53	0.28	0.518	0.451	0.239
		2020	0.51	0.31			
	Genistein 7-*O*-(2″-*O*-apiosyl)glucoside (F5)	2019	1.40	1.50	0.363	0.065	0.974
		2020	1.05	1.18			
	Genistein 7-*O*-glucoside (genistin) (F6)	2019	19.75	8.40	<0.001	<0.001	0.237
		2020	14.19	7.20			
	Daidzein 4′-*O*-(6″-*O*-malonyl)glucoside (F7)	2019	6.66	2.97	0.026	0.723	0.360
		2020	5.39	2.31			
	Genistein 5-*O*-(6″-*O*-malonyl)glucoside (F8)	2019	1.76	1.72	0.994	0.219	0.885
		2020	1.91	1.81			
	Daidzein 7-*O*-(4″-*O*-malonyl)glucoside (4″-*O*-malonyldaidzin) (F9)	2019	1.11	0.62	<0.001	0.321	0.352
		2020	0.64	0.29			
	Glycitein 4′-*O*-(6″-*O*-malonyl)glucoside (F10)	2019	0.86	0.46	0.828	0.257	0.346
		2020	0.85	0.40			
	Daidzein 7-*O*-(6″-*O*-malonyl)glucoside (6″-*O*-malonyldaidzin) (F11)	2019	75.39	32.16	0.069	0.873	0.325
		2020	66.94	26.89			
	Glycitein 7-*O*-(6″-*O*-malonyl)glucoside (6″-*O*-malonylglycitin) (F12)	2019	10.18	5.98	0.457	0.229	0.527
		2020	9.25	5.20			
	Genistein 4′-*O*-(6″-*O*-malonyl)glucoside (F13)	2019	9.78	3.14	<0.001	0.008	0.474
		2020	6.99	3.14			
	Genistein 7-*O*-(4″-*O*-malonyl)glucoside (4″-*O*-malonylgenistin) (F14)	2019	1.84	0.67	<0.001	0.955	0.274
		2020	1.10	0.40			
	Genistein 7-*O*-(6″-*O*-malonyl)glucoside (6″-*O*-malonylgenistin) (F15)	2019	134.71	41.78	0.019	0.004	0.505
		2020	116.05	43.28			
	Daidzein (F16)	2019	0.77	0.50	<0.001	0.425	0.904
		2020	0.31	0.21			
	Glycitein (F17)	2019	0.16	0.24	0.288	0.314	0.752
		2020	0.07	0.22			
	Genistein 7-*O*-(6″-*O*-acetyl)glucoside (6″-*O*-acetylgenistin) (F18)	2019	0.29	0.51	0.748	0.319	0.809
		2020	0.23	0.67			
	Genistein (F19)	2019	0.76	0.37	0.185	0.064	0.113
		2020	0.55	0.29			
	Daidzein group	2019	96.73	41.91	0.036	0.928	0.316
		2020	82.59	33.57			
	Genistein group	2019	171.17	53.57	0.004	0.003	0.393
		2020	142.85	53.73			
	Glycitein group	2019	15.88	9.28	0.544	0.224	0.457
		2020	14.63	8.13			
	Total (F20)	2019	283.78	89.76	0.010	0.156	0.336
		2020	240.07	85.78			

^(1)^*p* value by two-way ANOVA.

## Data Availability

Not applicable.
